# Case Report: Tyrosine kinase inhibitors in ROS1-rearranged and granulocyte colony-stimulating factor-producing lung squamous cell carcinoma

**DOI:** 10.3389/fonc.2026.1933312

**Published:** 2026-08-25

**Authors:** Ruijie Li, Shengjie Zhang, Zhenghe Liu, Xuan Wu

**Affiliations:** Department of Medical Oncology, Henan Provincial Chest Hospital, Chest Hospital of Zhengzhou University, Zhengzhou, Henan, China

**Keywords:** case report, entrectinib, granulocyte colony-stimulating factor, ROS1 fusion, squamous cell carcinoma

## Abstract

**Background:**

c-ros oncogene 1 (ROS1) fusions occur in less than 0.2% of lung squamous cell carcinomas (SqCCs). Concurrent production of granulocyte colony-stimulating factor (G-CSF) induces marked leukocytosis and is associated with aggressive tumor behavior and resistance to conventional therapies. Here, we report a case with this rare and aggressive dual-pathology subtype response to tyrosine kinase inhibitors.

**Case presentation:**

A 53-year-old male smoker with stage IVb SqCC (cT3N3M1) presented marked leukocytosis, and elevated serum G-CSF. The disease progressed following platinum-doublet chemotherapy and immunotherapy, despite a programmed death ligand 1 (PD-L1) tumor proportion score of 50% or greater. Next-generation sequencing revealed an Ezrin (EZR)-ROS1 fusion. Initiation of crizotinib induced rapid partial response and leukocytosis normalization for 6 months. Sequential lorlatinib achieved sustained disease control for 10 months. Due to economic considerations, entrectinib was initiated and that maintained over 15-month ongoing response with minimal toxicity of grade 1 anemia.

**Conclusion:**

This report suggests ROS1 fusion, G-CSF-producing lung SqCC could achieve a favorable response to molecularly-guided therapy with ROS1-tyrosine kinase inhibitors, highlighting a potential therapeutic strategy for this rare subgroup.

## Introduction

c-ros oncogene 1 (ROS1) fusions can promote tumor growth through constitutively activating downstream pathways (1, 2). The ROS1 oncogene, first identified in the UR2 avian sarcoma virus, maps to chromosome 6q21 and encodes a 2347-amino acid tyrosine kinase receptor with unknown physiological ligands (17). ROS1 fusions occur in 1-2% of non-small cell lung cancer (NSCLC), predominantly affecting young, never-smoking Asian patients with adenocarcinoma and are exceedingly rare (less than 0.2%) in lung squamous cell carcinoma (SqCC) (3–5). Notably, SqCC harboring ROS1 fusions is typically negative for canonical adenocarcinoma markers [Thyroid Transcription Factor-1 (TTF-1) negativity], and the clinicopathological distinctiveness remains poorly characterized due to the extreme rarity of these tumors (6, 7). Granulocyte colony-stimulating factor (G-CSF) production is a rare paraneoplastic syndrome, characterized by aggressive disease biology, marked leukocytosis, and a poor prognosis (8–12). The co-occurrence of ROS1 fusion and G-CSF production in SqCC represents a rare clinicopathological entity, posing significant diagnostic and therapeutic challenges.

The advent of ROS1 tyrosine kinase inhibitors (TKIs), such as crizotinib and lorlatinib, have revolutionized the management for ROS1-driven malignancies (13–15). Entrectinib, a next-generation ROS1-TKI, achieves therapeutic cerebro-spinal fluid concentrations, translating to superior intracranial activity (16).

Herein, we report a male smoker with metastatic G-CSF-producing lung SqCC harboring an Ezrin (EZR)-ROS1 fusion. The patient achieved sustained systemic response following sequential treatment with crizotinib, lorlatinib, and entrectinib, accompanied by normalization of serum G-CSF levels.

## Case presentation

**Figure 1:**
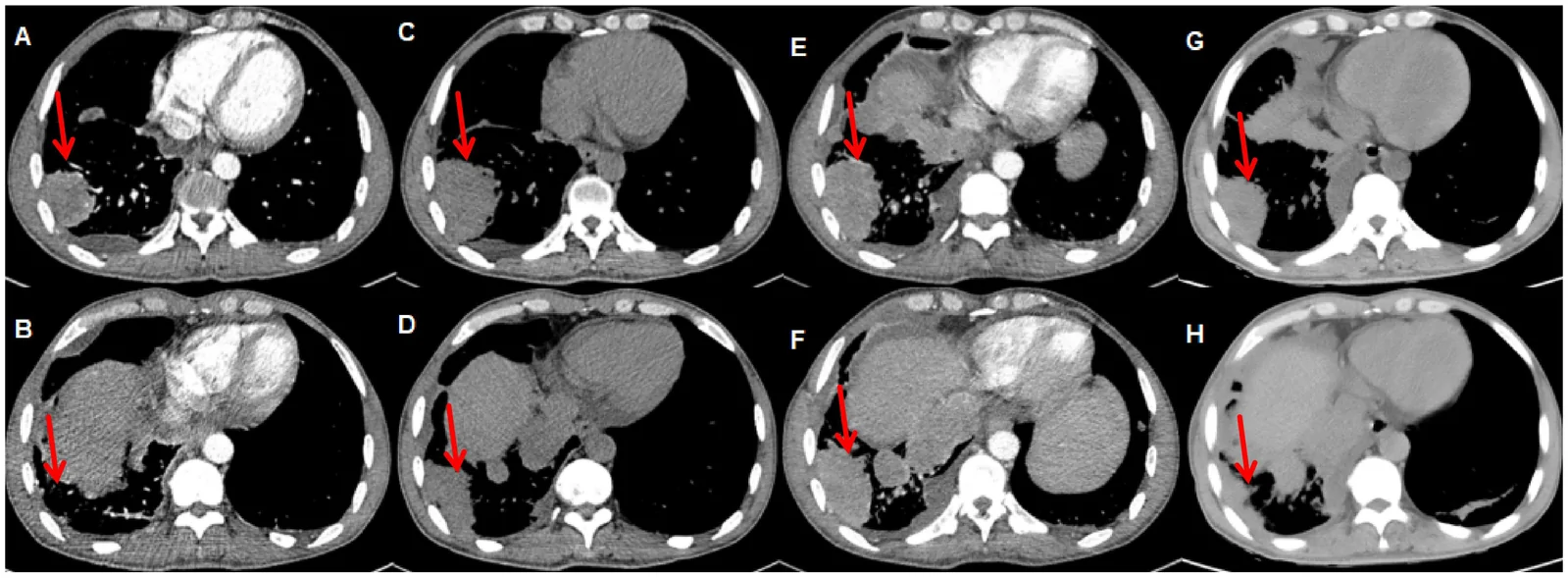
Serial chest CT images during the disease course and therapeutic response. Before treatment **(A, B)**, the target lesion was a 43×33 mm right lower lobe mass, with non-target lesions including pleural nodules and lymphadenopathy. After one cycle of gemcitabine, nedaplatin, and camrelizumab **(C, D)**, the target lesion increased to 62×40 mm (Progressive Disease). Following one cycle of liposomal paclitaxel and nedaplatin plus camrelizumab **(E, F)**, further progression to 62×45 mm was observed (Progressive Disease). After two cycles of liposomal paclitaxel/cisplatin/endostar **(G, H)**, the target lesion was 50×33 mm with a new left lower lobe lesion (Progressive Disease).

A 53-year-old male with a smoking history presented to our hospital on June 26, 2022, with progressive chest tightness, dyspnea, and exertional breathlessness. Contrast-enhanced chest computed tomography (CT) revealed a 43×33 mm pleural-based mass in the right lower lobe, enlarged right hilar and mediastinal lymph nodes. Laboratory studies demonstrated marked leukocytosis (32.87×10^9^/L) with neutrophilia (30.79×10^9^/L), elevated serum G-CSF (75.44 pg/mL), hypoglycemia (glucose 1.71 mmol/L), and elevated serum squamous cell carcinoma antigen (SCCA) (80.186 ng/mL). A CT-guided biopsy established a diagnosis of moderately differentiated SqCC (**Figures 1A, B**), with positive CK5/6/p40/p63, negative TTF-1/Napsin A, and programmed death ligand 1 (PD-L1) expression of 80% (tumor proportion score) (**Supplementary Figure 1**). The patient was diagnosed with stage IVb (cT3N3M1) SqCC with G-CSF production.

**Figure 2:**
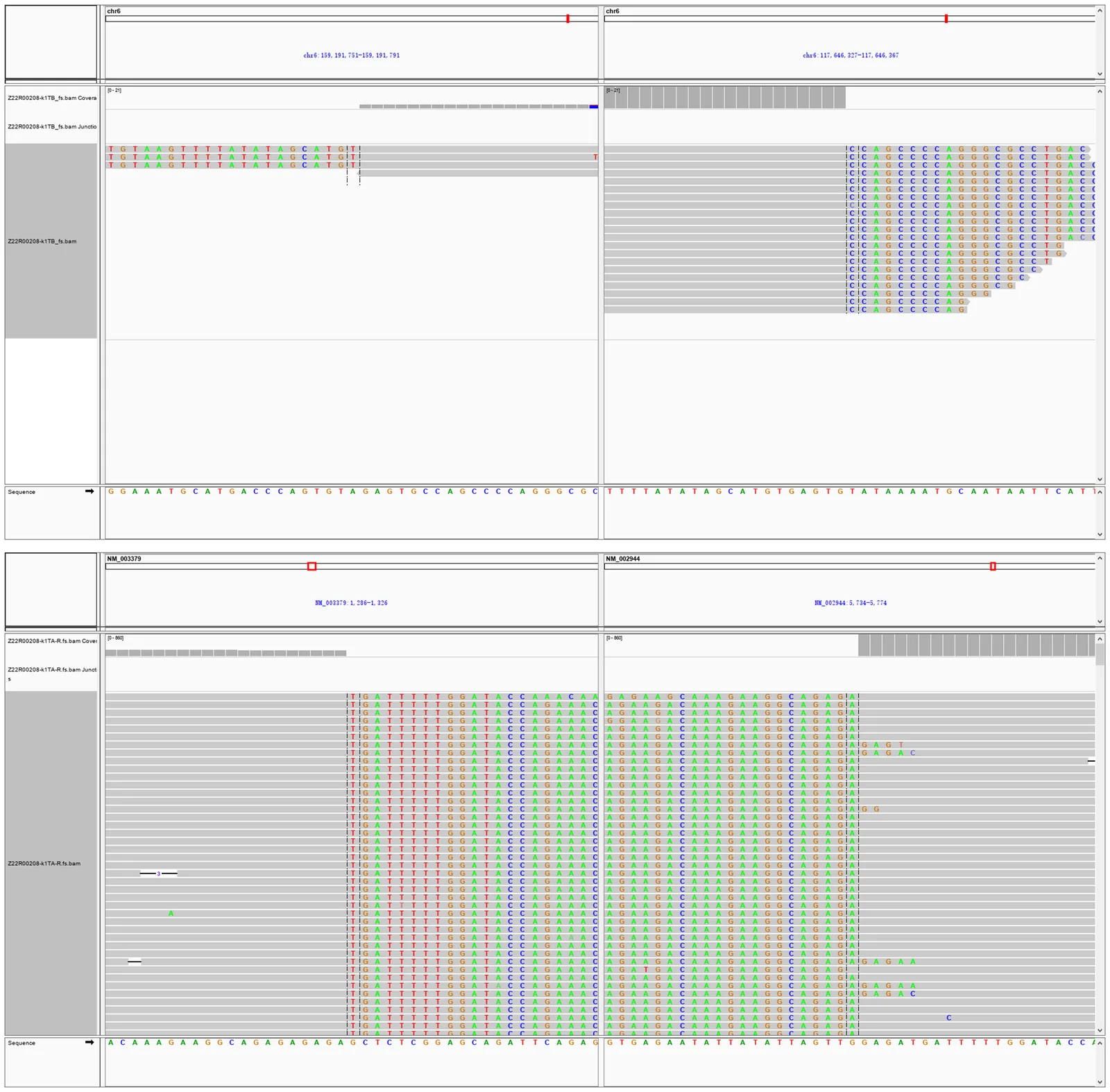
Sequencing reads of the EZR and ROS1 are shown by the Integrative Genomics Viewer. Gene-level schematic showing the EZR-ROS1 fusion breakpoint. NGS analysis identified an in-frame fusion between EZR exon 9 (chr6:159,191,771) and ROS1 exon 34 (chr6:117,646,347).

Initial therapy with gemcitabine, nedaplatin, and camrelizumab (July 6, 2022) resulted in progressive disease (by means of Response Evaluation Criteria in Solid Tumors version 1.1) (**Figures 1C, D**). Given this initial failure, a subsequent cycle of liposomal paclitaxel and nedaplatin plus camrelizumab (**Figures 1E, F**) and two cycles of liposomal paclitaxel/cisplatin/endostar (August-September 2022), similarly proved ineffective, with persistent disease progression accompanied by recalcitrant leukocytosis even up to 160×10^9^/L (**Figures 1G, H**). The striking refractoriness to both chemotherapy and immunotherapy, combined with the paraneoplastic hematologic derangement, prompted us to perform comprehensive molecular profiling of the initial biopsy specimen via next-generation sequencing (NGS), which identified an EZR-ROS1 fusion (allele fraction 30.77%) (**Figure 2**). The fusion involved EZR exon 9 fused in-frame to ROS1 exon 34, preserving the intact ROS1 tyrosine kinase domain.

**Figure 3:**
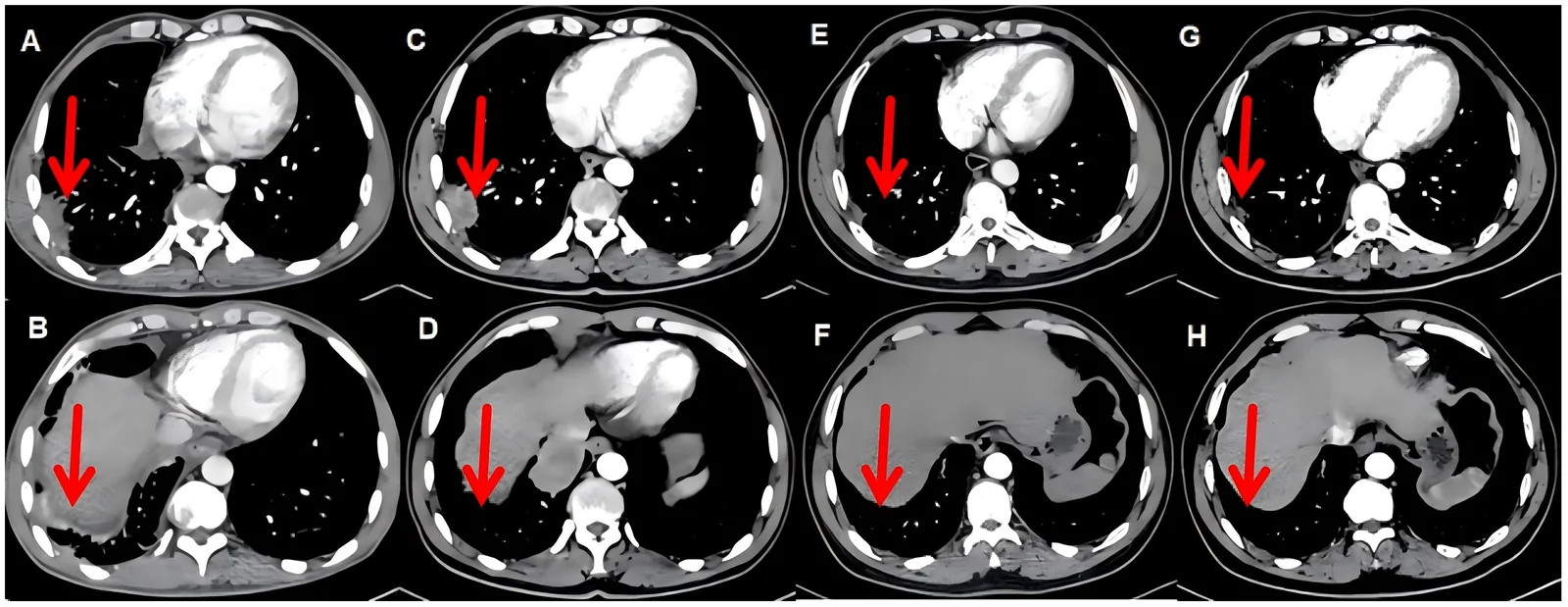
Serial chest CT scans during the disease course and therapeutic response. **(A, B)** One month after oral crizotinib (Partial Response). **(C, D)** After 6 months of crizotinib treatment (Progressive Disease). **(E, F)** After 10 months of lorlatinib treatment (Partial Response). **(G, H)** 15 months after entrectinib treatment (Partial Response).

Given the patient’s demonstrated resistance to multiple platinum-based chemotherapy and immunotherapy regimens, which was further exacerbated by G-CSF-mediated myeloproliferation and its potential immunosuppressive effects, we attempted targeted inhibition of the identified driver oncogene. Crizotinib (250 mg twice daily) was initiated on September 20, 2022. Within one month, CT demonstrated a partial response (**Figures 3A, B**), which was maintained for 6 months until disease progression on April 13, 2023 (**Figures 3C, D**). Subsequently, a repeat lung biopsy was performed for pathological and molecular analysis, which confirmed the persistent presence of the same fusion mutation without detectable resistance mutations. Based on this finding and the known efficacy of next-generation TKIs. Then, lorlatinib (100 mg daily) was initiated and achieved a secondary partial response sustained for 10 months (**Figures 3E, F**). In March 2024, entrectinib was added to the National Reimbursement Drug List in China. Given this improved accessibility, coupled with the patient’s financial constraints regarding lorlatinib, we transitioned treatment to entrectinib (600 mg daily). At the time of transition, the patient had achieved stable disease on lorlatinib with no evidence of radiographic progression. The decision to switch to entrectinib was based on the patient’s financial constraints and the recent inclusion of entrectinib in the National Reimbursement Drug List, which made it more affordable, whereas lorlatinib remained out-of-pocket. Sustained disease control has been observed on entrectinib at the last follow-up on June 15, 2025, representing an ongoing response exceeding 15 months (**Figures 3G, H**). Additionally, the white blood cell (WBC) counts were closely monitored during the entire treatment period, and the dynamic changes are depicted in **Supplementary Figure 2**.

## Discussion

This case report describes a 53-year-old male smoker with stage IVb lung SqCC and G-CSF production-a rare dual-pathology entity that posed significant therapeutic challenges. We first discuss the clinical rarity and demographic distinctiveness of this presentation, then examine the biological role of G-CSF in driving tumor aggressiveness and immunotherapy resistance, followed by an analysis of the TKI response patterns observed in our patient, and finally address the clinical implications for molecular profiling in refractory SqCC.

Despite high PD-L1 expression, disease progressed on platinum-based chemotherapy and immunotherapy. Identification of an EZR-ROS1 fusion prompted crizotinib therapy, inducing rapid partial response and normalization of paraneoplastic leukocytosis. Sequential treatment with crizotinib, lorlatinib, and entrectinib achieved sustained disease control, demonstrating efficacy of ROS1 inhibition in this molecularly distinct SqCC subset.

The present case diverges demographically as a male smoker with SqCC. Additionally, G-CSF production-first described by Asano et al. in 1977-manifests as paraneoplastic neutrophilia without infection (17).

G-CSF producing tumors are diagnosed based on elevated serum G-CSF, positive immunohistochemical staining, and reversible leukocytosis upon tumor regression, after excluding infectious etiologies. G-CSF-producing tumors predominantly affect older males and are associated with a poor prognosis (8, 9). Although they can occur in various histologies, G-CSF gene expression is more frequent in squamous cell carcinoma (33.3%) than in adenocarcinoma (6%) (18). G-CSF production has been reported in lung cancers harboring EGFR mutations (19) and ALK rearrangements (20), where is associated with TKI resistance. Beyond the role in granulopoiesis, tumor-derived G-CSF fosters an immunosuppressive microenvironment by recruiting myeloid-derived suppressor cells (MDSCs), which inhibit T-cell function. This mechanism underlies the aggressive behavior, poor prognosis, and therapeutic resistance-including to immunotherapy-observed across multiple cancer types (8). Moreover, The review by Karagiannidis et al. provides a comprehensive framework for understanding how G-CSF shapes an immunosuppressive tumor microenvironment (8). The authors demonstrate that G-CSF promotes the expansion of myeloid-derived suppressor cells and drives macrophage polarization toward a pro-tumorigenic M2 phenotype. Most relevant to our case, they specifically show that G-CSF reduces CD4+ and CD8+ T cell infiltration into tumors and that this directly correlates with reduced efficacy of PD-1/PDL-1 blockade immunotherapy. Furthermore, they highlight STAT3 as a central mediator of these effects, noting that G-CSF signals through STAT3 and that STAT3 in turn transcriptionally activates G-CSF in a feed-forward loop.

Here, extreme leukocytosis (WBC>30×10^9^/L) correlated with tumor burden, resolved during TKI response, and recurred at progression, satisfying diagnostic criteria alongside elevated serum G-CSF (75.44 pg/mL) (**Supplementary Table 1**). G-CSF-producing tumors typically exhibit platinum resistance and poor immunotherapy responses-consistent with treatment trajectory in this case (10, 21).

Notably, the patient had artifactual hypoglycemia (glucose 1.71 mmol/L) caused by *in vitro* glycolysis due to extreme leukocytosis. Whole-blood glucose declined over time, while serum glucose remained stable-a phenomenon attributed to glucose consumption by leukocytes. Crizotinib induced rapid response, though PFS was shorter than in ROS1-rearranged adenocarcinomas (PROFILE 1001: median PFS 19.3 months; OO-1201: 15.9 months (13–15). This may reflect G-CSF-associated aggressiveness or squamous biology, yet TKI tolerance was exceptional (only grade 1 anemia vs. typical visual/GI toxicities). To our knowledge, this represents the first reported G-CSF-producing ROS1-rearranged SqCC responsive to sequential TKIs. The exceeding 15-month ongoing response to entrectinib underscores the durability of ROS1 inhibition in such rare subsets.

G-CSF secretion drives paraneoplastic neutrophilia and systemic inflammation, classically conferring platinum resistance and immunotherapy refractoriness as observed here (10, 21, 22). Our patient (PD-L1 TPS 80%, progressed on anti-PD-1) directly mirrors Miyazaki et al., who reported that 69.2% of G-CSF-producing lung cancers had PD-L1 ≥50%, yet pembrolizumab response rate was only 33.3% (23). This suggests G-CSF production may override high PD-L1 expression as a determinant of immunotherapy response. Karagiannidis et al. document that G-CSF promotes MDSC expansion via STAT3, M2 macrophage polarization, and reduced CD4^+^/CD8^+^ T cell infiltration, leading to reduced efficacy of PD-1/PDL-1 blockade immunotherapy (8). In our patient, extreme leukocytosis (WBC 160×10^9^/L) likely reflects MDSC expansion, creating an immunosuppressive niche explaining immunotherapy resistance. Moreover, the exceeding 15-month ongoing response to entrectinib aligns with median PFS 19.3 months from PROFILE 1001 data for ROS1-adenocarcinomas, suggesting the superior ROS1 inhibition overcoming squamous-specific kinase constraints and resistance mutations. Furthermore, the rapid normalization of leukocyte counts (WBC: 32.4 to 7.1 ×10^9^/L within two weeks) likely attenuated G-CSF-driven immunosuppression, potentially reversing the expansion of myeloid-derived suppressor cells-a key mediator of T-cell dysfunction in this PD-L1-high tumor (14, 24). The artifactually low whole-blood glucose (1.71 mmol/L) further exemplifies leukocyte-mediated ex vivo glycolysis, necessitating serum glucose verification in extreme leukocytosis. This sustained response, achieved with minimal toxicity (grade 1 anemia only), supports the use of expanded molecular profiling in patients with SqCC who present with paraneoplastic syndromes.

This case offers *in vivo* confirmation of EZR-ROS1 as a functional driver in SqCC, a fusion-prone histology often overlooked for targeted therapy. The concomitant normalization of paraneoplastic leukocytosis upon TKI initiation underscores successful suppression of ROS1-mediated downstream signaling, highlighting the dual efficacy of targeted inhibition against both tumor growth and its systemic sequelae. These observations advocate for broader genomic profiling in refractory SqCC, especially when atypical clinical presentations point to non-canonical oncogenic drivers.

## Conclusion

This case demonstrates that ROS1 fusions, though rare in SqCC, can serve as actionable drivers conferring sustained response to sequential TKIs-even in G-CSF producing tumors refractory to chemotherapy and immunotherapy.

## Data Availability

The original contributions presented in the study are included in the article/**Supplementary Material**. Further inquiries can be directed to the corresponding authors.
