# Supplementary material for: Case Report: Tyrosine kinase inhibitors in ROS1-rearranged and granulocyte colony-stimulating factor-producing lung squamous cell carcinoma
Source: Front Oncol. 2026 Aug 25;16:1933312. doi: 10.3389/fonc.2026.1933312 (PMC13549892; doi:10.3389/fonc.2026.1933312)
Supplement: Supplementary file 1 [file SupplementaryFile1.docx]

**Supplemental Figure 1.** White blood cell counts during the entire treatment period.

**Supplemental Figure 2.** Immunohistochemistry for CK5/6 (A), p40 (B), p63 (C), and TTF-1 (D) in the tumor.
