# Supplementary material for: Case Report: Tyrosine kinase inhibitors in ROS1-rearranged and granulocyte colony-stimulating factor-producing lung squamous cell carcinoma
Source: Front Oncol. 2026 Aug 25;16:1933312. doi: 10.3389/fonc.2026.1933312 (PMC13549892; doi:10.3389/fonc.2026.1933312)
Supplement: Supplementary file 2 [file Table1.docx]

**Supplemental Table 1.** Summary of clinical parameters, treatment, and radiological response over the disease course.

| **Time point** | **WBC (×10⁹/L)** | **Serum G-CSF (pg/mL)** | **SCCA (ng/mL)** | **Glucose (mmol/L)** | **Target lesion size (mm)** | **Treatment** | **Radiological response** |
| --- | --- | --- | --- | --- | --- | --- | --- |
| Baseline (2022-06-26) | 32.87 | 75.44 | 80.186 | 1.71 | 43×33 | — | — |
| Post-chemo/immuno (2022-08) | up to 160 | N/A | N/A | N/A | 62×45 | Gemcitabine + nedaplatin + camrelizumab; liposomal paclitaxel + nedaplatin + camrelizumab; liposomal paclitaxel/cisplatin/endostar | PD |
| 1 month post-crizotinib (2022-10) | 7.1 (normalized) | N/A | N/A | N/A | 39×28 (PR) | Crizotinib | PR |
| Progression post-crizotinib (2023-04-13) | elevated (recurred) | N/A | N/A | N/A | 55×38 | Crizotinib | PD |
| 10 months post-lorlatinib (2024-02) | Normal | N/A | N/A | N/A | 38×25 (PR) | Lorlatinib | PR |
| Last follow-up (2025-06-15) | Normal | N/A | N/A | N/A | 35×22 (PR) | Entrectinib | PR (ongoing >15 months) |

Abbreviations: WBC, white blood cell; G-CSF, granulocyte colony-stimulating factor; SCCA, squamous cell carcinoma antigen; PR, partial response; PD, progressive disease; N/A, not available at that time point.
